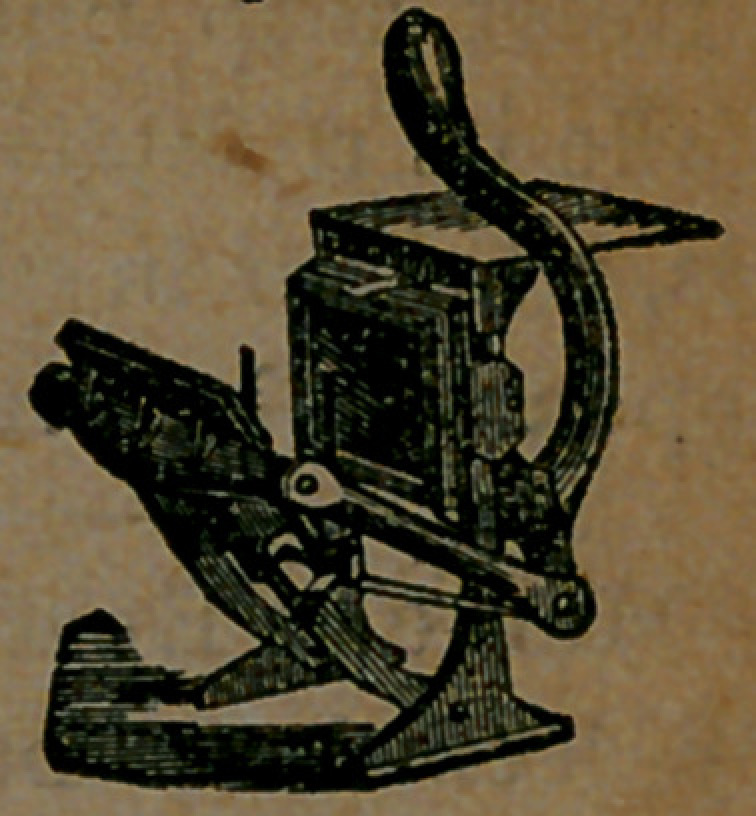# The Art Preservative

**Published:** 1875-07

**Authors:** 


### THE ART PRESERVATIVE.

The Art of Printing appears to be rapidly becoming an accomplishment for young people of both sexes, its mysteries being no longer confined to those who have served a prolonged period of apprenticeship for their acquisition. This result has been brought about by the introduction of small presses of varying excellence, on some of which as good work can be produced, as on any of the large and costly machines.

We have long thought that a discussion of this subject in our pages, would be likely to accomplish a useful purpose. We see in these little presses, a chance for boys and girls to employ themselves very pleasantly, and even profitably, while at the same time, acquiring nearly as much practical knowledge as the average school would afford them.

Some time ago we received a long letter from a smart boy of thirteen years, in which he recited his own experiences with various styles of presses, relating his failures to secure a clean and perfect impression from his types on the presses which he first used, and his gratifying success on a press of another make. He thought we ought to tell all the boys and girls that the machine which he called "The Young America Press," was the only useful press to be had at a reasonable price. We did not think we would be justified in this, until we had accumulated other testimony as to the merits of the various presses designed for the use of amateurs. So we looked up the evidence, and are satisfied that our young friend was right. We are able to find no other low-priced press which will compare in efficiency with the Young America Press. This is the uniform testimony with all whom we have met, who have used this and other presses.

It is powerful and strong, as will be seen by reference to this little wood-cut, which we have prepared in order that that our readers may better understand its construction. This is a view of the Note-Press, the "form" or type of which, when set, will measure five by seven and a half inchcs. Its weight is fifty pounds, and its cost is $18. The work done on this little press is as perfect, we think, as can be done on any press in the world, if care is taken.

There are many other sizes and styles of Young America presses, made, as is the one we have shown, by Mr. Joseph Watson of No. 73 Cornhill Street, Boston, and No. 53 Murray Street, New York; but this size is probably the one which most boys and girls would prefer to own. We can think of no present which would prove as attractive and valuable, to a bright boy or girl, as this press, and a little lot of type; and as a means of education, it is evident that nothing more effective can be employed. The most admirable of "object lessons" are thus given; by which spelling and punctuation, and the construction of sentences and proof-reading, are practically taught. The instructions which are sent out with each press, enables any child to commence printing at once, and in a week or two, the work may be made a source of profit, in the way of card and circular printing for others. We will allude to this subject again; but would meanwhile advise our friends to write to Mr. Watson as above, for his descriptive circulars.

## Figures and Tables

**Figure f1:**